# Effect of cover crops on the yield and nutrient concentration of organic kale (*Brassica oleracea* L. var. *acephala*)

**DOI:** 10.1038/s41598-019-46847-9

**Published:** 2019-07-17

**Authors:** Dil Thavarajah, Niroshan Siva, Nathan Johnson, Rebecca McGee, Pushparajah Thavarajah

**Affiliations:** 1 Plant and Environmental Sciences, 270 Poole Agricultural Center, Clemson University, Clemson, SC 29634 USA; 2 USDA Agriculture Research Service, Grain Legume Genetics and Physiology Unit, Washington State University, Pullman, WA 99164-6434 USA

**Keywords:** Plant sciences, Plant physiology

## Abstract

Kale is a leafy green vegetable regularly grown using non-organic agricultural systems. In recent years, organic kale demand has increased at near-doubling rates in the USA due to its perceived nutritional value. The objective of this study was to determine the effect of organic cover cropping systems on subsequent kale biomass production and nutrient composition (protein, mineral, and prebiotic carbohydrate concentrations) and to assess organic kale as a potential whole food source of daily essential mineral micronutrients and prebiotic carbohydrates. A single 100-g serving of fresh organic kale can provide mineral micronutrients (43–438 mg Ca; 11–60 mg Mg; 28–102 mg P; 0.5–3.3 mg Fe; 0.3–1.3 mg Mn; 1–136 µg Cu; and 0–35 µg Se) as well as 5.7–8.7 g of total prebiotic carbohydrates, including sugar alcohols (0.4–6.6 mg), simple sugars (6–1507 mg), raffinose and fructooligosaccharides (0.8–169 mg), hemicellulose (77–763 mg), lignin (0–90 mg), and unknown dietary fiber (5–6 g). Fresh organic kale has low to moderate concentrations of protein (1.3–6.0 g/100 g). Study results indicate that Starbor and Red Russian are the most suitable kale cultivars for organic production without considerable biomass and nutrient composition losses. Among the cover crops, faba bean results in the highest mineral, protein, and prebiotic carbohydrate concentrations in subsequent kale crops but ryegrass increases kale biomass production. Results also demonstrated a significant interaction between kale variety and organic cover crop with respect to biomass and nutrient concentration. Future organic nutritional breeding of kale is possible by selecting cultivars that perform well following different cover crops.

## Introduction

Originating from the eastern Mediterranean, kale is one of the oldest leafy brassica vegetables. Kale was not introduced to the USA until the early 1980s^1^, but since then has been marketed in grocery stores as different varieties and at various stages of maturity, packed as fresh food ready to eat or cook. Kale is ranked 15^th^ on the list of “powerhouse” fruits and vegetables (a 100-g serving provides ≥10% of the daily value of 17 essential nutrients)^2^. These “powerhouse” fruits and vegetables are strongly associated with reduced risk of heart disease and other non-communicable diseases^2^. Consequently, kale is gaining popularity in communities around the world but remains an understudied vegetable, especially in terms of organic production.

Since the early 1990s, American consumer demand for organic vegetables has significantly increased^3^. Today, the US organic vegetable industry is worth approximately $29 billion annually and is growing as a result of human health concerns, such as breast cancer, liver disease, and heavy metal toxicity^3,4^. Organic produce also plays a predominant role in detoxification and obesity prevention therapies^4^. However, a primary criticism of organic agriculture is lower production and nutrient composition compared to non-organic systems^5^. US-certified organic agriculture production has increased since the introduction of the Organic Foods Production Act of 1990^6^. The United States Department of Agriculture (USDA) National Organic Standards Board defines organic agriculture as “an ecological production management system that promotes and enhances biodiversity, biological cycles, and soil biological activity^6^”. Currently, USDA statistics are not available for organic kale production and utilization, but it is an emerging cash crop for many small-scale organic producers in the southern USA. Therefore, comprehensive nutrient composition data and knowledge regarding ways to increase organic kale production will enable kale breeders to incorporate nutritional quality traits in their breeding programs to develop nutrient-rich kale targeted for organic production.

Studies comparing the nutritional quality of organic kale with other leafy vegetables are uncommon^3,7–10^. Organic farming systems generally provide a range of soil, biological, ecological, and other environmental benefits^11–14^. Consumer concerns regarding the nutritional quality and chemical safety of foods are considered to be the main reasons for the increasing demand for organic produce, which consumers perceive as healthier and safer^15,16^. Cover crops have been used for centuries to provide multiple ecological benefits to both organic and non-organic cropping systems, such as preventing soil erosion, improving physical and biological properties of the soil, supplying nutrients [nitrogen (N) and phosphorus (P)], suppressing weeds, increasing soil water retention, and breaking pest/disease cycles^17^. Legume cover crops are widespread in organic cropping systems as a result of symbiotic biological N fixation and subsequent soil N benefits, especially for leafy vegetables^17^. Organic fertilizers and animal manure are other options for organic vegetable production. However, heavy metal uptake and food safety issues are significant concerns for organic leafy vegetable production using animal waste from meat processing or chicken or swine manure^18^. Therefore, N fixation from legume cover crops is a safer alternative for subsequent vegetable crops. N fixation quantities are dependent on the legume species, growth stage, length of growing season, climate, and soil conditions^19^. To our knowledge, no studies have endeavored to understand the influence of organic cover crop on subsequent kale production and nutritional quality. We hypothesize that fall planting of legume cover crops including faba bean, winter field pea, hairy vetch, crimson clover, and ryegrass (as a control) will affect subsequent spring organic kale biomass production and nutritional composition. The objectives of this study were therefore to determine how different organic cover cropping systems [faba bean (FB); Windham winter pea (WWP); hairy vetch (HV); Lynx winter pea (LWP); crimson clover (CC); and ryegrass (RG)] affect subsequent kale biomass production and nutrient composition (protein, mineral, and prebiotic carbohydrate concentrations), and to assess organic kale as a potential whole food source of daily essential minerals and prebiotic carbohydrates.

## Results

Organic kale was found to be moderate in protein (1.3–6.0 g/100 g), essential minerals, and prebiotic carbohydrates (5.7–8.7 g/100 g). Types and concentrations of these essential minerals and prebiotic carbohydrates in organic kale were variable **(**Table 1**)**. The total identified prebiotic carbohydrates in organic kale ranged from 0.7 to 2.7 g/100 g; however, 5.0 to 6.0 g/100 g of unidentified soluble prebiotic carbohydrates were found **(**Table 1**)**. Kale cultivar, cover crop, and the interaction between cultivar and cover crop treatment were significant in most cases for prebiotic carbohydrates and minerals **(**Table 2**)**. The nutrient quality of kale cultivars varied in response to different organic cover crops (Supplementary Data Table 1). Organic kale nutritional quality might be maximized through ideal cover crop and kale cultivar pairing; the cultivar by organic cover crop treatment was significant (*P* < *0.05*) for protein, minerals (excluding Fe and Cu), and prebiotic carbohydrates (excluding mannitol and nystose) **(**Table 2**)**. Overall, these organic kale cultivars differ in terms of prebiotic carbohydrates, minerals, protein, and biomass.

**Table 1:** Range and mean nutrient concentrations (protein, minerals, and prebiotic carbohydrates) of organic kale grown in SC.

Nutrient	Organic kale^a^	
	Range	Mean
Protein (g/100 g)	1.3–6.0	3.7
***Minerals (mg/100 g)***		
K	108–410	241
Ca	43–438	204
Mg	11–60	29
P	28–102	60
Fe	0.5–3.3	1.0
Zn	0.2–0.7	0.3
Mn	0.3–1.3	0.6
Cu (µg/100 g)	1–136	47
Se (µg/100 g)	ND^b^ −35	8.0
***Prebiotic carbohydrates (mg/100 g)***		
***Sugar alcohols***		
Sorbitol	1.1–6.6	2.2
Mannitol	ND- 0.4	0.1
***Simple Sugars***		
Glucose	80–982	434
Fructose	401–1507	976
Sucrose	6–318	38
Mannose	11–46	26
***RFO and FOS***		
Sta + Raf	13–169	73
Ver + Kes	0.8–39	13
Nystose	ND −10	1.0
***Hemicellulose***		
Arabinose	ND -763	417
Xylose	ND -77	25
***Lignin***	0–90	19
Total known prebiotic carbohydrates (g/100 g)	0.7–2.7	1.9
Unknown prebiotic carbohydrates (g/100 g)	5.0–6.0	4.5
Total prebiotic carbohydrates (g/100 g)	5.7–8.7	6.4

^a^Values based on the combined statistical analysis of 108 data points for the current study (fresh weight, 85% moisture). ^b^ND, not detectable levels.

**Table 2:** Analysis of variance of prebiotic carbohydrate and mineral concentrations for kale cultivars grown in organic cover crops.

Source	df	Sugar Alcohols		Simple Sugars				RFO + FOS			Hemicellulose		Lignin	TP
		Sor	Man	Glu	Fru	Suc	Mann	Sta + Raf	Ver + Kes	Nys	Ara	Xyl		
Cultivar	5	**	**	**	**	**	**	**	**	**	**	**	**	**
Cover crop	5	**	*	**	**	**	**	**	**	**	**	**	**	**
Replication	2	NS	NS	NS	NS	NS	NS	NS	NS	NS	NS	NS	NS	NS
Cultivar × cover crop	25	**	NS	**	**	**	**	**	**	NS	**	**	NS	**
Error	107													
	df	K	Ca	Mg	P	Fe			Zn	Mn		Cu		Se
Cultivar	5	**	**	**	**	**			**	**		NS		**
Cover crop	5	**	**	**	**	*			**	**		**		**
Replication	2	NS	NS	NS	NS	NS			NS	NS		NS		NS
Cultivar × cover crop	25	**	**	**	**	NS			*	**		NS		**
Error	107													

Sorbitol (Sor), Mannitol (Man), Glucose (Glu), Fructose (Fru), Sucrose (Suc), Mannose (Mann), Stachyose and Raffinose (Sta + Raf), Verbascose and Kestose (Ver + Kes), Nystose (Nys), Arabinose (Ara), Xylose (Xyl), Total prebiotic carbohydrates (TP).

**Significant at *P* < *0.05*, *significant at *P* < *0.1*.

**Figure 1:**
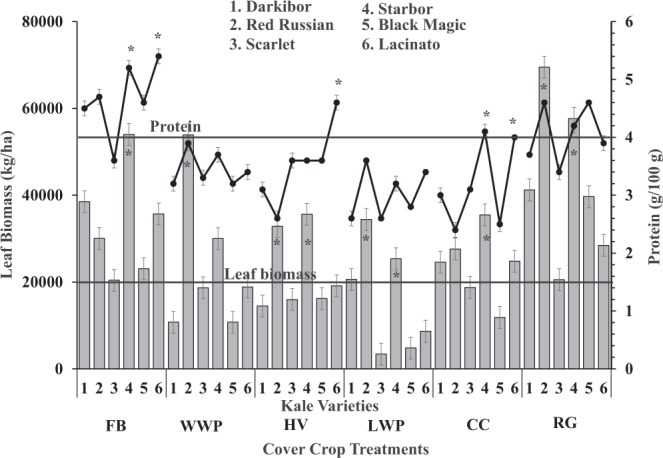
Variation of leaf biomass and protein content of six kale cultivars grown in six different organic cover cropping systems. *leaf biomass and protein within varieties for different cover crops are significantly different at p < 0.05.

A 100-g serving of fresh organic kale can provide up to 44% of the recommended daily allowance (RDA) for Ca, 14–19% (male/female) for Mg, 18–41% (female/male) for Fe, 57–72% (male/female) for Mn, 15% for Cu, and 64% for Se **(**Table 3**)**. Protein content and leaf biomass were compared between the six kale cultivars for each of the six cover cropping systems **(**Fig. 1**)**. Starbor yielded significantly higher (P < 0.05) protein following faba bean and crimson clover, while cv Lacinato protein was significantly higher following faba bean, hairy vetch, and crimson clover covers. Red Russian yielded significantly higher leaf biomass following Windham winter pea, Lynx winter pea, and ryegrass covers. Starbor and Lacinato showed higher protein concentration in most cases than the other varieties **(**Fig. 1**)**.

**Table 3:** %RDA of different minerals in a 100-g serving of fresh organic kale grown in SC, USA.

Element	%RDA from 100 g^a^	Recommendation
K	2.3–8.7	Male/Female (4.7 g)
Ca	4.3–44	Male/Female (1 g)
Mg	2.6–14	Male (400–420 mg)
	3.4–19	Female (310–320 mg)
P	4.0–15	Male/Female (700 mg)
Fe	6.3–41	Male (8 mg)
	2.8–18	Female (18 mg)
Zn	1.8–6.4	Male (11 mg)
	2.5–8.8	Female (8 mg)
Mn	13–57	Male (2.3 mg)
	17–72	Female (1.8 mg)
Cu	0.1–15	Male/Female (900 µg)
Se	0–64	Male/Female (55 µg)

^a^Percent recommended daily allowance (%RDA) was calculated based on National Academy of Sciences (2004)^33^. Values are based on fresh weight (85% moisture), n = 108 per nutrient.

A significant (P < 0.05) interaction was evident between different cover cropping systems and kale cultivars in terms of mean prebiotic carbohydrate concentrations **(**Table 4**)**. Kale grown following the crimson clover cover cropping system had the most prebiotic carbohydrate concentrations (9 of 12 carbohydrates) that were significantly higher than other cropping systems, followed by faba bean (6 of 12), hairy vetch (5 of 12), and Lynx winter pea (5 of 12) **(**Table 4**)**. The crimson clover cover cropping system yielded kale cultivars with RFO + FOS concentrations significantly higher than all other systems. Red Russian had the most prebiotic carbohydrate concentrations (5 of 12) that were significantly higher than other cultivars, followed by Darkibor (4 of 12) and Scarlet (4 of 12). Darkibor had significantly higher RFO concentrations than all other cultivars **(**Table 5**)**.

**Table 4:** Prebiotic carbohydrate levels in organic kale cultivars in response to different cover crops.

Cover Crop	Sugar Alcohols		Simple Sugars				RFO + FOS			Hemicellulose		Lignin
	Sor	Man	Glu	Fru	Suc	Mann	Sta + Raf	Ver + Kes	Nys	Ara	Xyl	
	mg/100 g											
FB	2.5 *a*	0.08 *a*	401 *b*	945 *b*	22 *c*	32 *a*	66 *bc*	9.4 *c*	1.5 *a*	551 *a*	28 *a*	15 *b*
WWP	1.6 *b*	0.03 *b*	402 *b*	866 *c*	36 *b*	20 *d*	67 *bc*	12.1 *b*	1.1 *b*	496 *b*	29 *a*	25 *a*
HV	2.2 *a*	0.05 *a*	447 *ab*	891 *c*	25 *c*	29 *a*	74 *bc*	13.3 *b*	0.5 *c*	423 *c*	29 *a*	20 *a*
LWP	2.3 *a*	0.09 *a*	466 *ab*	1097 *a*	82 *a*	20 *d*	62 *c*	10.0 *c*	0.8 *c*	469 *b*	26 *a*	15 *b*
CC	2.2 *a*	0.08 *a*	494 *a*	1025 *a*	42 *b*	28 *b*	91 *a*	23.5 *a*	1.4 *a*	405 *c*	24 *a*	26 *a*
RG	2.1 *a*	0.07 *a*	394 *b*	1030 *a*	22 *c*	25 *c*	77 *b*	11.5 *b*	0.9 *c*	156 *d*	12 *b*	11 *c*

Means within a column followed by different letters are significant at *P* < 0.05. Faba bean (FB); Windham winter pea (WWP); hairy vetch (HV); Lynx winter pea (LWP); crimson clover (CC); ryegrass (RG).

**Table 5:** Overall prebiotic carbohydrate concentrations of six different kale cultivars.

Variety	Sugar alcohols		Simple Sugars				RFO + FOS			Hemicellulose		Lignin
	Sor	Man	Glu	Fru	Suc	Mann	Sta + Raf	Ver + Kes	Nys	Ara	Xyl	
	mg/100 g											
Darkibor	1.9 *b*	0.02 *c*	507 *b*	957 *b*	37 *b*	22 *b*	113 *a*	18.0 *a*	1.0 *b*	497 *a*	30 *a*	33 *b*
Red Russian	1.8 *b*	0.12 *a*	629 *a*	1199 *a*	34 *b*	24 *b*	43 *c*	10.2 *c*	1.8 *a*	304 *c*	26 *a*	6.2 *c*
Scarlet	1.9 *b*	0.08 *b*	352 *d*	940 *b*	31 *b*	28 *a*	76 *b*	15.2 *b*	0.8 *b*	485 *a*	28 *a*	45 *a*
Starbor	2.4 *a*	0.06 *b*	372 *d*	964 *b*	19 *c*	25 *b*	79 *b*	13.1 *b*	0.6 *b*	400 *b*	23 *b*	14 *c*
Black Magic	2.4 *a*	0.04 *b*	447 *b*	955 *b*	85 *a*	26 *b*	71 *c*	14.0 *b*	1.0 *b*	416 *b*	23 *b*	7.8 *c*
Lacinato	2.5 *a*	0.06 *b*	298 *d*	840 *c*	22 *c*	30 *a*	54 *c*	9.1 *c*	0.8 *b*	397 *b*	19 *c*	6.4 *c*

Means in columns followed by different letters are significant at *P* < 0.05.

Different cover cropping systems and kale cultivars had significant (P < 0.05) effects on mean mineral concentrations. Kale grown following faba bean had the most mineral concentrations (7 of 9 minerals) that were significantly higher than for other cropping systems. Phosphorous and Mn concentrations were significantly higher in all kale cultivars following faba bean cover than all other systems. Hairy vetch, crimson clover, and ryegrass followed faba bean in terms of some mineral concentrations that were significantly higher than for other cropping systems (4 of 9) **(**Table 6**)**. Overall, Starbor had the most mineral concentrations (5 of 9) that were significantly higher than other varieties, followed by Lacinato (3 of 9) and Scarlet (2 of 9) **(**Table 7**)**.

**Table 6:** Mineral concentrations in organic kale cultivars in response to different cover crops.

Cover Crop	K	Ca	Mg	P	Fe	Zn	Mn	Cu	Se
	mg/100 g							µg/100 g	
FB	275 *a*	249 *a*	41 *a*	89 *a*	1.0 *a*	0.42 *a*	0.79 *a*	51 *b*	7.0 *b*
WWP	200 *b*	222 *a*	28 *b*	50 *b*	0.7 *b*	0.34 *a*	0.61 *b*	43 *b*	1.0 *c*
HV	222 *a*	272 *a*	34 *a*	44 *b*	1.2 *a*	0.20 *c*	0.61 *b*	43 *b*	2.7 *c*
LWP	191 *b*	100 *b*	16 *c*	46 *b*	0.7 *b*	0.28 *c*	0.58 *b*	16 *c*	2.3 *c*
CC	181 *b*	251 *a*	31 *a*	50 *b*	1.6 *a*	0.25 *c*	0.63 *b*	75 *a*	ND
RG	240 *a*	187 *b*	29 *b*	66 *b*	1.1 *a*	0.44 *a*	0.65 *b*	46 *b*	25 *a*

^a^Means within a column followed by different letters are significantly different at p < 0.05. Faba bean (FB); Windham winter pea (WWP); hairy vetch (HV); Lynex winter pea (LWP); crimson clover (CC); ryegrass (RG); not detectable levels (ND).

**Table 7:** Overall mineral concentrations in six different kale cultivars.

Variety	K	Ca	P	Mg	Fe	Mn	Zn	Cu	Se
	mg/100 g							µg/100 g	
Darkibor	263 *b*	203 *b*	46 *b*	23 *b*	1.0 *b*	0.60 *b*	0.22 *b*	66	ND
Red Russian	221 *b*	169 *b*	59 *b*	29 *b*	0.8 *b*	0.39 *c*	0.31 *a*	72	4.0
Scarlet	274 *b*	311 *a*	57 *b*	40 *a*	1.1 *b*	0.72 *b*	0.27 *b*	70	ND
Starbor	338 *a*	273 *b*	61 *a*	45 *a*	2.1 *a*	0.94 *a*	0.27 *b*	75	1.0
Black Magic	181 *c*	251 *b*	50 *b*	31 *b*	1.6 *b*	0.63 *b*	0.25 *b*	75	ND
Lacinato	262 *b*	287 *b*	66 *a*	31 *b*	1.4 *b*	0.87 *a*	0.36 *a*	63	ND

^a^Means within a column followed by different letters are significantly different at p < 0.05. ND, not detectable levels.

## Discussion

Organic cover cropping systems have a significant effect on subsequent kale biomass production and nutrient composition. Our study results clearly show that ryegrass is the most suitable cover crop for subsequent kale biomass production (42,846 kg/ha) followed by faba bean (33,624 kg/ha); the Lynex winter pea cover crop is the least suitable for subsequent organic kale production (16,224 kg/ha; Fig. 1). Similar results reported for annual ryegrass as a cover crop for subsequent vegetable production^20,21^ indicate 80% of the ryegrass biomass establishes within the first month of the growing period and provides greater carbon and N biomass quantities to subsequent vegetable crops than legume cover crops, which require temperatures below 15–18 °C and longer times^17^. Although grass cover crops establish faster than legumes, organic kale produced after faba bean cover had higher levels of protein (4.7 g/100 g), minerals (Ca, Mg, P, and Mn), and prebiotic carbohydrates (mannose, nystose, arabinose, xylose, and lignin) than kale produced after ryegrass cover **(**Tables 4 and 6; Fig. 1). Therefore, organic kale production is possible by optimizing the legume cover cropping system. However, further studies are required to confirm planting dates and cultivar effects on nutritional quality considering different organic farm locations and climatic regions.

The highest concentrations of K, P, Mg, Fe, and Mn were observed in the curly kale cultivar Starbor, followed by Lacinato for P, Mn, and Zn; Scarlet for Ca and Mg; and Red Russian for Zn **(**Table 7**)**. Further, these kale cultivars were also high in Cu; however, Se concentrations were below the detection limit for most cultivars except Red Russian and Starbor **(**Table 7**)**. Kale cultivars varied with respect to protein levels. Lacinato and Starbor had higher protein levels (4.0–4.1 g/100 g) than other cultivars. Prebiotic carbohydrates were particularly abundant in Darkibor, Red Russian, and Scarlet. Starbor and Lacinato were high in minerals that are under-consumed by Americans, making kale a low-calorie food^22^ that is a good source of essential minerals and prebiotic carbohydrates. Current organic kale production cultivars have been bred from non-organic production; therefore, they may not be well suited to organic production. For example, these varieties may have low biomass, production issues, and low nutritional quality (especially protein, prebiotic carbohydrate quality, and micronutrients) when grown under organic conditions. Therefore, further studies will be required to establish optimal organic kale breeding in the USA.

Data from this study provide evidence that organic kale, grown using different cover cropping systems, is a whole food source of the essential minerals Ca, Mg, P, Fe, Mn, Cu, and Se as well as significant levels of prebiotic carbohydrates **(**Table 1**)**. However, the absolute nutrient bioavailability of these minerals (Fe and Zn) from organic kale should be established using future feeding trials. Organic kale also provides moderate to low levels of protein. Kale appears to be a good source of minerals and prebiotic carbohydrates and could constitute a significant source of these nutrients in American diets, as per the recommendations of the 2015 Dietary Guidelines Advisory Committee^23^. Overall, fall-grown ryegrass and faba bean cover crops are the most suitable systems for increased biomass production and nutritional quality of subsequent spring organic kale production.

The health and nutritional sectors are paying increasing attention to organic kale. However, little research has been done to assess the nutritional value of organic kale. Data from the present study support the notion that organic kale can provide significant essential minerals and adequate prebiotic carbohydrates quantities with moderate to low levels of protein^22^. Other organic vegetable studies show that protein content increases with increased nitrogen supply, and soluble sugars and starches increase when soil P levels are generally lower than other soil nutrients^23–25^. Most organic vegetable producers in the southern USA use winter legume and grass mixes as cover crops to provide significant amounts of N, P, and K to subsequent vegetable crops, as well as to reduce soil erosion, nematode populations, and soil-borne diseases and to suppress weeds^20^. The current goals of US organic farming systems are to maintain soil fertility, avoid pollution, use crop rotations, protect animal welfare, and support environmental sustainability^14^. To these ends, it is essential within the organic farming framework to focus on organic plant breeding activities that will result in cultivars that are more suitable for organic production and will deliver in terms of enhanced nutritional quality and mineral bioavailability^5^.

In conclusion, organic cover cropping systems have a significant effect on subsequent kale biomass production as well as protein, essential minerals, and prebiotic carbohydrate concentrations. For kale biomass production, ryegrass is the most suitable cover crop followed by faba bean. Kale cultivars Lacinato and Starbor have high protein levels and Darkibor, Red Russian, and Scarlet are abundant in prebiotic carbohydrates and minerals. Further opportunities should be pursued to improve the nutritional and production values of organic kale as well as to provide more significant quantities of nutrients to kale consumers.

## Materials and Methods

### Materials

Standards, reagents, acids, and high-purity solvents used for kale sample and reagent preparation were purchased from Sigma-Aldrich Co. (St. Louis, MO, USA) and VWR International (Radnor, PA, USA) and used without further purification. Water, distilled and deionized (ddH_2_O) to resistance of ≥18.2 MΩ (Milli-Q Water System, Millipore, Milford, MA), was used for kale sample and reagent preparation.

### Field design

The experimental field design was a complete randomized block design with three replicates of six commercial kale cultivars grown in six cover cropping systems (n = 108; Table 8).

**Table 8:** Organic cover crops and kale cultivars used in this experiment.

Cover crop; seeding rate; seeding depth	1. Faba bean (FB); 100 lb/ac; 2.5 cm
	2. Windham winter pea (WWP); 100 lb/ac; 2.5 cm
	3. Hairy vetch (HV); 25 lb/ac; 1 cm
	4. Lynex winter pea (LWP); 100 lb/ac; 2.5 cm
	5. Crimson clover (CC); 20 lb/ac; 1 cm
	6. Ryegrass (RG); 110 lb/ac; 2.5 cm
Kale cultivars	▪ Curly kale: Darkibor, Red Russian, Scarlet, Starbor
	▪ Dinosaur kale: Black Magic, Lacinato
Replications	3
Total number (n)	108

### Land preparation

Organic field experiment was conducted at Clemson University, SC, USA. Certified organic seeds for cover crops faba bean (Windsor), crimson clover, hairy vetch, and ryegrass were obtained from Johnny’s Select Seeds, ME, USA. Seeds for the two winter field pea cultivars, Windham and Lynx, were obtained from the USDA-ARS field pea breeding program at Washington State University, WA (Table 8). Cover crop plants were established in the Clemson University USDA Certified Student Organic Farm in October 2016 using protocols and recommendations developed by the USDA Sustainable Agriculture Research and Education Program (Table 8**)**^26^. Each cover crop was planted using a small seed drill in 7 × 8 m plots at a seeding rate of 20–100 lb/ac with a seeding depth of 1–2 cm depending on the cover crop species (Table 7)^26^. No fertilizer or any organically certified chemicals were added to the cover crop plots. Weeds were manually removed every week, and cover crops were irrigated using a drip irrigation system (0.4 gal/h for 30 min at 8–9 am). Cover crops were crimped 15 days before kale transplanting, and the planting rows were prepared using a small tractor.

### Kale experiment

For kale transplants, organic kale seeds were grown in an organically certified poly house for three weeks, and then six kale cultivars with three replicates per row were planted in each cover crop plot on March 24, 2017 **(**Table 8**)**. Six commercially grown kale cultivars were selected based on market class, consumer demand, disease resistance, drought tolerance, biological yield, and our current breeding and selection research **(**Table 8**)**^22,27^. Weeds were removed manually every week, and plants were irrigated twice a day using a drip irrigation system (1.5 L/h for 30 min). No organic fertilizer was added. Field-planted kale seedlings became infested with fire ants during the first week of planting, which was controlled using two applications of organically certified *Bacillus thuringiensis* (B.t.; 1 mL/L). Kale plants were harvested at physiological maturity (ready to eat) in mid-May 2017. A single kale plant per replicate was harvested, the stem removed, and then the remainder weighed for leaf biomass production. Fresh kale leaf samples (250 g) were then taken randomly from the entire harvested plant of each of three independent replicated field plots and subjected to protein, mineral, and prebiotic carbohydrate analyses. A total of 108 replicate kale leaf samples were collected. The moisture content of fresh sub-samples (105 °C for 16 h) was determined, and the remaining samples immediately stored at −40 °C until analysis. Before each analysis, the oven-dried samples were finely ground using a mortar and pestle. Nutrient composition data are reported on a fresh weight basis (85% moisture).

### Protein analysis

Total nitrogen level was determined on oven-dried finely ground kale leaf samples using a LECO FP3000 CNS analyzer; protein content was calculated as N × 6.25^28^.

### Mineral concentrations

Mineral concentrations were measured by inductively coupled plasma atomic emission spectroscopy (ICP-AES) using a Thermo 6500 Duo instrument (Thermo Fisher Scientific, PA, USA) after modified HNO_3_-H_2_O_2_ digestion^29^. Aliquots (500 mg) of oven-dried samples were digested with 6 mL of 70% HNO_3_ overnight at room temperature. They were then heated to 90 °C for one h, after which 3 mL of 30% H_2_O_2_ were added and the sample held at 90 °C for 15–20 min. Finally, 3 mL of 6 M hydrochloric acid was added, and the samples held at 90 °C for another 5 min. Samples were then filtered and made up to 10 mL in Milli-Q water^29^. Detection limits were 30 μg/L for K, Ca, and Mg and 5 μg/L for Fe, Zn, Mn, Cu, and Se. Analytical quality assurance was accomplished using authentic calibration standards and the NIST standard reference material peach leaves 1547. The percentages of recommended daily allowance values (%RDA)^30^ for each mineral were calculated for a 100-g portion of fresh kale.

### Prebiotic carbohydrate concentrations

Oven-dried finely ground kale leaf samples (~500 mg) were extracted as per the method reported by Muir *et al*.^31^. This method involved extraction with distilled water at 80 °C for one h. The resulting extract was passed through a 13 mm × 0.45 µm nylon syringe filter (Chromatographic Specialties, Brockville, ON, Canada). Analyses for sugar alcohols, monosaccharides, disaccharides, and oligosaccharides were performed using high-performance liquid-liquid partition chromatography (ICS-5000 Dionex, Sunnyvale, CA, USA) as described by Feinberg^32^ with pulsed amperometric detection (PAD, Dionex) in series with electrochemical detection (Thermo Fisher Scientific, Inc.). Carbohydrates were separated by a CarboPac PA-100 4 × 250 mm column in series with a CarboPac PA-100 4 × 50 mm guard column (Dionex, Sunnyvale, CA, USA). The mobile phase flow rate was maintained at 1 mL/min. Solvents used for elution were 100 mM sodium hydroxide/600 mM sodium acetate (solvent A), 200 mM sodium hydroxide (solvent B), and ddH_2_O (solvent C). The initial 16 min used 50% B and 50% C, followed by a gradient change to 3% A, 48.5% B, and 48.5% C. At 18 min, the gradient changed linearly to 16% A, 42% B, and 42% C. Finally, at 19 min, the gradient returned to 50% B and 50% C. The silver-silver chloride electrode was set at 2.0 μA. The minimum detectable level (signal to noise ratio ≥3) of each analyte was 0.1 ppm. Peak areas for a reference lentil sample were routinely analyzed with an error of less than 5%, and linear calibration models for standards also had errors <5%. Concentrations of carbohydrates in the filtrate (C) were determined from the calibration model used to calculate concentrations in sample dry matter in the expression X = (C × V)/m, where X is the concentration of the carbohydrate in the sample (corrected for moisture), V is the final diluted volume, and m is the mass of the dry sample aliquot. Hemicellulose was measured as described above after digesting a 500-mg sample with 5 mL of 7% (w/w) hydrochloric acid (HCl) at 55 °C for 120 min^22^. Lignin was determined using an aliquot (0.9 mL) of extract added into a cuvette with 0.1 mL of neutral ferric chloride (5 g in 100 mL) solution, the absorbance of which was then measured at 690 nm in a spectrophotometer (GENESYS 20, Thermo Scientific, Ashville, NC, USA). The concentration of lignin (equivalent to gallic acid) was measured using a standard curve. All data are presented as mg/100 g fresh weight.

### Statistical analysis

Data from the complete randomized block design represented three replicates of six kale cultivars grown in six cover cropping systems (n = 108). Replicates and cultivars were considered as random factors. Class variables include cultivar, replication, and cover cropping treatment. A mixed model analysis of variance was performed using the PROC GLM procedure of SAS version 9.4^33^. Means were separated by Fisher’s protected least significant difference at *P* < 0.05.

## Supplementary information

Supplementary Dataset 1

## References

[1] Balkaya A, Yanmaz R (2005). Promising kale (*Brassica oleracea* var. *acephala*) populations from the Black Sea region, Turkey. New Zeal J Crop Hortic Sci..

[2] Di Noia J (2014). Defining powerhouse fruits and vegetables: a nutrient density approach. Prev Chronic Dis..

[3] USDA ERS - Organic Market Overview. 2018. https://www.ers.usda.gov/topics/natural-resources-environment/organic-agriculture/organic-market-overview.aspx (accessed 02 Sep. 2018).

[4] Crinnion W (2010). Organic foods contain higher levels of certain nutrients, lower levels of pesticides, and may provide health benefits for the consumer. Altern Med Rev..

[5] Trewavas A (2001). Urban myths of organic farming. Nature.

[6] USDA- Organic Production/Organic Food: Information Access Tools. 2018. https://www.nal.usda.gov/afsic/organic-productionorganic-food-information-access-tools#define (accessed 02 Sep. 2018).

[7] Kawashima L, Soares L (2003). Mineral profile of raw and cooked leafy vegetables consumed in southern Brazil. J Food Compos Anal..

[8] Ayaz F (2006). Nutrient contents of kale (*Brassica oleraceae* L. var. *acephala* DC.). Food Chem..

[9] Lefsrud MG, Kopsell DA, Kopsell DE, Curran-Celentano J (2006). Irradiance levels affect growth parameters and carotenoid pigments in kale and spinach grown in a controlled environment. Physiol Plant..

[10] Fadigas JC (2010). Use of multivariate analysis techniques for the characterization of analytical results for the determination of the mineral composition of kale. Microchem J..

[11] Reganold JP, Glover JD, Andrews PK, Hinman HR (2001). Sustainability of three apple production systems. Nature.

[12] Bulluck Iii LR, Brosius M, Evanylo GK, Ristaino JB (2002). Organic and synthetic fertility amendments influence soil microbial, physical and chemical properties on organic and conventional farms. Appl Soil Ecol..

[13] Kramer SB, Reganold JP, Glover JD, Bohannan BJ, Mooney HA (2006). Reduced nitrate leaching and enhanced denitrifier activity and efficiency in organically fertilized soils. Proc Natl Acad Sci..

[14] Murphy KM, Campbell KG, Lyon SR, Jones SS (2007). Evidence of varietal adaptation to organic farming systems. F Crop Res..

[15] Magkos F, Arvaniti F, Zampelas A (2006). Organic food: buying more safety or just peace of mind? A critical review of the literature. Crit Rev Food Sci Nutr..

[16] Williamson CS (2007). Is organic food better for our health?. Nutr Bull..

[17] Snapp SS (2005). Evaluating cover crops for benefits, costs, and performance within cropping system niches. Agron J..

[18] Cang L (2004). Heavy metals pollution in poultry and livestock feeds and manures under intensive farming in Jiangsu Province, China. J Environ Sci..

[19] Shennan C (1992). Cover crops, nitrogen cycling, and soil properties in semi-irrigated vegetable production systems. HortScience.

[20] Creamer NG, Baldwin KR (2000). An evaluation of summer cover crops for use in vegetable production systems in North Carolina. HortScience.

[21] Creamer NG, Bennett MA, Stinner BR (1997). Evaluation of cover crop mixtures for use in vegetable production systems. HortScience.

[22] Thavarajah D (2016). Mineral micronutrient and prebiotic carbohydrate profiles of USA-grown kale (*Brassica oleracea* L. var. *acephala*). J Food Compos Anal..

[23] Heldt HW (1977). Role of orthophosphate and other factors in the regulation of starch formation in leaves and isolated chloroplasts. Plant Physiol..

[24] Locascio, S. J., Wiltbank, W. J., Gull, D. D. & Maynard, D. N. Fruit and vegetable quality as affected by nitrogen nutrition. *Nitrogen Crop Prod*. 617–626 (1984).

[25] Brandt K, Mølgaard JP (2001). Organic agriculture: does it enhance or reduce the nutritional value of plant foods?. J Sci Food Agric..

[26] Sustainable Agriculture Research and Education Program - Grants and Education. 2018. https://www.sare.org/ (accessed 02 Sep. 2018).

[27] Pathirana P (2017). Moisture deficit effects on kale (*Brassica oleracea* L. var. *acephala*) biomass, mineral, and low molecular weight carbohydrate concentrations. Sci Hortic..

[28] AOAC. Official Methods of Analysis. 17th ed. Association of Official Analytical Chemists, Washington, DC (2000).

[29] Thavarajah D, Thavarajah P, Sarker A, Vandenberg A (2009). Lentils (*Lens culinaris* Medikus Subspecies *culinaris*): a whole food for increased iron and zinc intake. J Agric Food Chem..

[30] Institute of Medicine of the National Academies. Dietary Reference Intakes for Energy, Carbohydrate, Fiber, Fat, Fatty Acids, Cholesterol, Protein, and Amino Acids (Macronutrients). National Academies Press: Washington, DC, (2005).

[31] Muir JG (2009). Measurement of short-chain carbohydrates in common Australian vegetables and fruits by high-performance liquid chromatography (HPLC). J Agric Food Chem..

[32] Feinberg M, San-Redon J, Assié A (2009). Determination of complex polysaccharides by HPAE-PAD in foods: validation using accuracy profile. J Chromatogr B.

[33] SAS Institute Inc. User’s guide: Statistics SAS Institute, Version 9.4. Cary, NC (2013).

