# Supplementary material for: Effect of cover crops on the yield and nutrient concentration of organic kale (Brassica oleracea L. var. acephala)
Source: Sci Rep. 2019 Jul 17;9:10374. doi: 10.1038/s41598-019-46847-9 (PMC6637178; doi:10.1038/s41598-019-46847-9)
Supplement: Supplementary file 1 — Supplementary Dataset 1 [file 41598_2019_46847_MOESM1_ESM.docx]

**Effect of cover crops on the yield and nutrient concentration of organic kale (*Brassica oleracea* L. var. *acephala*)**

Dil Thavarajah^1*^, Niroshan Siva^1^, Nathan Johnson^1^, Rebecca McGee^2^, Pushparajah Thavarajah^1^

^1^Plant and Environmental Sciences, 270 Poole Agricultural Center, Clemson University, Clemson, SC 29634, USA.

^2^USDA Agriculture Research Service, Grain Legume Genetics and Physiology Unit, Washington State University, Pullman, WA 99164-6434, USA.

^*^Author to whom correspondence should be addressed: e-mail address: dthavar@clemson.edu; Tel: +18646562607; Fax: +18646564960

**Supplementary Table 1**: Prebiotic carbohydrate concentrations of kale cultivars response to cover crops

| Cover Crop | Cultivar | Sugar alcohols^a^ | | Simple Sugars | | | | RFO+FOS | | | Hemicellulose | | Lignin | TP  (g/100 g) |
| --- | --- | --- | --- | --- | --- | --- | --- | --- | --- | --- | --- | --- | --- | --- |
|  |  | Sor | Man | Glu | Fru | Suc | Mann | Sta+Raf | Ver+Kes | Nys | Ara | Xyl |  |  |
|  |  | Mg/100g | | | | | | | | | | | |  |
| FB | Darkibor | 1.7 b | 0.06 | 440 | 906 b | 21 | 27 b | 96 a | 9.7 b | 0.4 | 601 a | 29 b | 29 a | 2.2 |
|  | Red Russian | 1.4 b | 0.13 | 542 | 1257 a | 26 | 27 b | 36 c | 5.4 c | 4.1 | 381 b | 40 a | 4 c | 2.3 |
|  | Scarlet | 2.2 b | 0.12 | 334 | 972 b | 22 | 36 a | 76 b | 14.4 a | 0.9 | 595 a | 25 c | 36 a | 2.1 |
|  | Starbor | 4.1 a | 0.03 | 288 | 843 b | 19 | 29 b | 82 b | 13.5 a | 0.6 | 636 a | 31 b | 15 b | 2.0 |
|  | Black Magic | 2.4 b | 0.05 | 475 | 787 b | 25 | 34 a | 46 c | 2.9 c | 1.9 | 608 a | 28 b | 9 c | 2.0 |
|  | Lacinato | 3.1 b | 0.10 | 325 | 904 b | 20 | 37 a | 62 b | 10.7 b | 1 | 486 b | 14 c | 1 c | 1.9 |
| WWP | Darkibor | 1.3 d | 0.01 b | 435 | 720 c | 33 ab | 15 b | 89 a | 15.5 a | 0.4 b | 425 b | 42 | 40 b | 1.8 ab |
|  | Red Russian | 1.3 d | 0.12 a | 481 | 1001 a | 28 ab | 21 b | 30 b | 6.4 b | 2.0 a | 350 c | 18 | 17 c | 2.0 ab |
|  | Scarlet | 1.5 c | 0.03 b | 414 | 876 b | 49 ab | 20 b | 78 a | 14.1 a | 1.3 b | 657 a | 33 | 62 a | 2.2 a |
|  | Starbor | 2.1 a | 0.02 b | 374 | 922 b | 18 b | 20 b | 75 a | 13.2 a | 0.8 b | 618 a | 30 | 21 c | 2.1 ab |
|  | Black Magic | 1.7 b | 0.01 b | 352 | 751 c | 53 a | 18 b | 61 a | 8.7 b | 0.8 b | 397 c | 22 | 26 c | 1.7 b |
|  | Lacinato | 2.0 a | 0.00 b | 357 | 927 b | 33 ab | 27 a | 68 a | 14.4 a | 1.1 b | 529 b | 32 | 11 c | 2.0 ab |
| HV | Darkibor | 2.5 | 0.02 | 551 b | 966 a | 29 b | 24 c | 122 a | 17.1 b | 1.1 a | 584 a | 37 a | 38 a | 2.4 a |
|  | Red Russian | 2.2 | 0.06 | 745 a | 994 a | 44 a | 20 c | 47 d | 12.7 c | 0.2 c | 290 c | 31 a | 12 b | 2.2 a |
|  | Scarlet | 1.9 | 0.05 | 283 d | 750 b | 15 c | 32 a | 55 c | 13.3 c | 0.2 c | 536 a | 40 a | 44 a | 1.8 b |
|  | Starbor | 1.8 | 0.03 | 374 c | 872 b | 16 c | 26 b | 80 b | 12.6 c | 0.5 b | 267 d | 16 b | 13 b | 1.7 c |
|  | Black Magic | 2.6 | 0.08 | 507 b | 1154 a | 39 a | 34 a | 106 a | 23.4 a | 0.2 c | 484 b | 37 a | 10 b | 2.4 a |
|  | Lacinato | 2.1 | 0.05 | 223 d | 610 c | 6 c | 36 a | 31 d | 0.9 d | 0.6 b | 378 b | 15 b | 9 b | 1.3 d |
| LWP | Darkibor | 1.7 c | 0.03 | 589 a | 1049 b | 64 b | 16 | 99 a | 19.1 a | 0.3 d | 588 a | 21 c | 30 a | 2.5 a |
|  | Red Russian | 1.7 c | 0.14 | 634 a | 1299 a | 28 c | 25 | 40 b | 1.6 d | 1.5 a | 347 b | 25 b | 5 b | 2.4 a |
|  | Scarlet | 2.1 b | 0.11 | 407 b | 1085 b | 52 b | 20 | 72 a | 17.9 a | 0.6 c | 622 a | 34 a | 40 a | 2.4 a |
|  | Starbor | 1.6 c | 0.05 | 536 a | 1212 a | 19 c | 17 | 64 b | 10.1 b | 0.8 b | 405 b | 38 a | 11 b | 2.3 a |
|  | Black Magic | 3.7 a | 0.04 | 331 b | 991 c | 301 a | 20 | 56 b | 6.0 c | 0.6 c | 441 b | 19 c | 2 b | 2.2 a |
|  | Lacinato | 3.2 a | 0.15 | 299 c | 948 c | 30 c | 24 | 43 b | 5.0 c | 0.9 b | 414 b | 21 c | 7 b | 1.8 b |
| CC | Darkibor | 2.1 | 0.02 b | 495 b | 971 b | 55 a | 28 a | 130 a | 29.0 a | 2.4 a | 666 a | 41 a | 42 b | 2.5 a |
|  | Red Russian | 2.1 | 0.11 b | 904 a | 1440 a | 58 a | 21 b | 77 c | 31.3 a | 1.6 a | 262 c | 30 a | 18 c | 2.8 a |
|  | Scarlet | 2.1 | 0.09 b | 426 b | 1010 b | 32 b | 33 a | 111 a | 22.8 b | 1.3 a | 434 b | 24 b | 59 a | 2.2 b |
|  | Starbor | 2.4 | 0.21 a | 245 c | 885 b | 19 c | 34 a | 77 c | 16.2 b | 0.5 b | 343 b | 18 b | 9 c | 1.7 c |
|  | Black Magic | 2.1 | 0.01 b | 644 b | 1141 a | 66 a | 20 b | 90 b | 30.3 a | 2.0 a | 330 b | 19 b | 20 c | 2.4 a |
|  | Lacinato | 2.5 | 0.02 b | 251 c | 701 c | 21 c | 30 a | 58 d | 11.2 c | 0.4 b | 393 b | 13 c | 18 c | 1.5 d |
| RG | Darkibor | 2.1 a | 0.02 b | 531 a | 1131 a | 30 a | 23 b | 143 a | 17.7 a | 1.4 a | 120 a | 8 b | 21 a | 2.0 a |
|  | Red Russian | 2.1 a | 0.18 a | 467 a | 1202 a | 22 a | 29 a | 29 d | 3.9 c | 1.5 a | 195 a | 12 b | 0 d | 2.0 a |
|  | Sacrlet | 1.8 b | 0.06 b | 246 c | 945 b | 17 b | 25 a | 62 c | 9.0 b | 0.7 b | 68 b | 11 b | 29 a | 1.4 d |
|  | Starbor | 2.2 a | 0.03 b | 417 a | 1048 a | 20 a | 22 c | 94 b | 12.8 b | 0.6 b | 135 a | 7 b | 12 b | 1.8 a |
|  | Black Magic | 2.1 a | 0.05 b | 372 b | 906 b | 24 a | 27 a | 70 c | 12.9 b | 0.6 b | 233 a | 12 b | 4 c | 1.7 b |
|  | Lacinato | 2.2 a | 0.04 b | 332 b | 946 b | 20 a | 23 b | 65 c | 12.5 b | 1.0 b | 183 a | 22 a | 3 c | 1.6 c |

Sorbitol (Sor), Mannitol (Man), Glucose (Glu), Fructose (Fru), Sucrose (Suc), Mannose (Mann), Stachyose and Raffinose (Sta+Raf), Verbascose and Kestose (Ver+Kes), Nystose (Nys), Arabinose (Ara), Xylose (Xyl), Total prebiotic carbohydrates (TP)

^a^ Means within a column followed by different letters are significantly different at p < 0.05.
